# Veratrum Viride, as a Poison

**Published:** 1852-07

**Authors:** E. M. Pendleton


*Veratrum Viride, as a Poison.* By E. M. Pendleton, M. D.—Having recently had the opportunity of trying the Tinc. of Veratrum Viride, (Dr. Norwood's preparation, purchased from himself, personally,) I feel it my duty to give a brief account of its therapeutical effects, as I would gladly have done, had the impressions made upon my mind been of a more favorable character. Ever loth to try new and potent remedies, as I abominate *experimentalism* in medicine, where danger may be apprehended, I had thus far resisted the temptation of using the article in question, notwithstanding the high character it had attained in the hands of Dr. Norwood and others, as has been made known in the Medical Journals. On the day in question, however, Dr. Norwood called at my office, and presented such a "cloud of witnesses," in the shape of letters received from divers physicians, some of whom I knew, that I was induced to take an ounce of his preparation, promising to give him, or some of the journals, the result of my experiments.

At noon of the same day, I called to see a patient laboring under Catarrhal Fever, whom I had bled in the morning to syncope, having had a remarkably hard and intractable pulse. She had been sick about a week, having the usual symptoms of Catarrhal or Inflammatory Fever—soreness in the muscles, pain in the back and head, red, watery eyes, and cough, with mucous expectoration. I found her pulse had rallied again, being very hard and bouuding, and going at the rate of 133 beats to the minute. A first rate case, thought I, for the Veratrum Viride. Accordingly, I prescribed Dr. Norwood's minimum dose, eight drops, pouring it out with my own hands, leaving directions for her to take another portion in three hours, the time designated by the Doctor.

About six, P. M., two hours after she had taken the last dose, I was sent for in great haste to see my patient, who was thought to be *moribund.* I was at her bed-side in a short time, and found her laboring under excruciating pain in the precordia, with a sense of suffocation and sinking at the stomach, cramps throughout the muscular system, which assumed a periodical character, a preturnatural coldness of the whole system, with clammy sudor. Her eyes were wild, pupils dilated, countenance expressive of great pain and apprehension, which were evidenced by her plaintive cries for help. She sometimes spoke incoherently, showiug a wandering mind, and a state bordering on the destruction of nervous vitality. Her pulse was scarcely perceptible and numbered 120 to the minute. I of course felt great alarm, and set to work to keep up the sinking powers, by warm mustard poultices and frictions externally, with wine and brandy internally. Notwithstanding all my efforts, the pulse became entirely imperceptible, and all the symptoms above described assumed a more alarming aspect, in connection with copious watery evacuations *per anum,* I ordered starch and laudanum injections, and persisted in the stimulants, etc., until about 8, P. M., two hours after my arrival, when her pulse again became perceptible, which was so weak that it reminded me more of the delicate thumping of the fetal heart on the tympanum in a stethoscopic examination four months after conception, than any thing else. What was remarkable, however, it was only 84 beats to the minute, showing at once the paternity of all this suffering and danger, as well as the potency of the drug administered, "for evil, however, rather than good." All the other unpleasant and deathly symptoms began to give way, as the heart which had been so suddenly crushed in its nervous energies, resumed its wonted sway. None but the truly conscientious physician, who holds himself accountable, not merely at the bar of public opinion, but at the bar of God, for all his professional, as well as other deeds, can appreciate my feelings at this termination of the case.

I found my patient, early the next morning, greatly relieved, pulse 96, full and strong. It continued to increase in frequency and strength until it reached nearly the condition it sustained prior to the administration of the Veratrum Viride. I of course resorted to another and safer class of remedies for its subduction, which resulted in a favorable termination of the case.

I have thus, in all candor, presented the results of my experience with a drug, whose sedative and poisonous qualities I regard but little if any inferior to the Prussic Acid, and decidedly more potent than the Digitalis. It is true only one case is presented, but one so clear and well marked, that none can attribute the result to other causes than the drug itself. Whatever effect the relation of this case may have upon the uninitiated, I must confess upon me it has had the effect to make me abandon the remedy, at least for the present. Bolder practitioners may, in this, as in other instances, educe good from evil, and free the remedy from some of its more poisonous principles; but our present knowledge of it will not warrant an indiscriminate use of it, without rendering us culpable of a violation of some of the plain laws of humanity and professional propriety, not to say of religion. —*Charleston Med. Journal.*

